# B-Cell-Based and Soluble Biomarkers in Body Liquids for Predicting Acute/Chronic Graft-versus-Host Disease after Allogeneic Hematopoietic Stem Cell Transplantation

**DOI:** 10.3389/fimmu.2016.00660

**Published:** 2017-01-16

**Authors:** Mateja Kralj Juric, Maxim Shevtsov, Petra Mozes, Justyna Ogonek, Rachel E. Crossland, Anne M. Dickinson, Hildegard T. Greinix, Ernst Holler, Eva M. Weissinger, Gabriele Multhoff

**Affiliations:** ^1^Department of Internal Medicine I, BMT, Medical University of Vienna, Vienna, Austria; ^2^Department of Radiation Oncology, Klinikum rechts der Isar, Technische Universität München, Munich, Germany; ^3^Department of Hematology, Hemostasis, Oncology and Stem Cell Transplantation, Transplantation Biology, Hannover Medical School, Hannover, Germany; ^4^Hematological Sciences, Institute of Cellular Medicine, Newcastle University, Newcastle upon Tyne, UK; ^5^Division of Hematology, Medical University of Graz, Graz, Austria; ^6^Department of Internal Medicine III, University Hospital of Regensburg, Regensburg, Germany

**Keywords:** biomarkers, graft-versus-host disease, proteomics, genomics, cellular, heat shock protein, B-cell subsets

## Abstract

Allogeneic hematopoietic stem cell transplantation (allo-HSCT) is the main curative therapy for hematological malignancy such as leukemias, lymphomas, or multiple myelomas and some other hematological disorders. In this therapy, cure of hematological diseases relies on graft-versus-malignancy effects by allogenic immune cells. However, severe posttransplant treatment-associated complications such as acute graft-versus-host disease (aGvHD) and chronic graft-versus-host disease (cGvHD) limit this approach. Most research into GvHD has concentrated on the aGvHD, while the more complex and multifaceted chronic form has been largely poorly investigated. cGvHD is a multi-organ autoimmune disorder and is the major cause of non-relapse morbidity and mortality following allo-HSCT, occurring in about 50% of patients, or 13,000–15,000 patients per year worldwide. Therefore, there is a high medical need for an early prediction of these therapy-associated toxicities. Biomarkers have gained importance over the last decade in diagnosis, in prognosis, and in prediction of pending diseases or side effects. Biomarkers can be cells, factors isolated from target tissues, or soluble factors that can be detected in body fluids. In this review, we aim to summarize some of the recent developments of biomarkers in the field of allo-HSCT. We will focus on cell-based biomarkers (B-cell subsets) for cGvHD and soluble factors including microRNA (miRNA), which are excreted into serum/plasma and urine. We also discuss the potential role of cytosolic and extracellular 70 kDa heat shock proteins (HSP70) as potential biomarkers for aGvHD and their role in preclinical models. Proteomic biomarkers in the blood have been used as predictors of treatment responses in patients with aGvHD for many years. More recently, miRNAs have been found to serve as a biomarker to diagnose aGvHD in the plasma. Another development relates to urine-based biomarkers that are usually detected by capillary electrophoresis and mass spectrometry. These biomarkers have the potential to predict the development of severe aGvHD (grades III–IV), overall mortality, and the pending development of cGvHD in patients posttransplant.

## Complications of Hematopoietic Stem Cell Transplantation (HSCT)

Acute and chronic graft-versus-host disease (a/cGvHD) are serious and frequent complications after allogeneic hematopoietic stem cell transplantation (allo-HSCT) that negatively impact on survival and quality of life of patients (1, 2). GvHD develops in approximately 40–60% of recipients after allo-HSCT. Donor-derived T-cells targeting alloantigens of the recipient play a key role in the induction of GvHD. Donor T-cells generally destroy host tissues by secreting pro-inflammatory cytokines such as interleukin-1 (IL-1), tumor necrosis factor alpha (TNF-α), and interferon gamma (IFN-γ) or by direct cytolytic activities of immune effector cells (1, 2). So far, GvHD is diagnosed based on unique diagnostic clinical signs and symptoms, as recommended by the National Institutes of Health (NIH) consensus development conferences (3, 4). In 2014, the NIH Consensus Conference updated requirements for the integration of assessment of potential biomarkers in prospective clinical studies of GvHD (4). Biomarkers that could be obtained by minimal invasive methods would be beneficial to predict GvHD and thus would increase safety and quality of life of patients. Biomarkers should fulfill certain requirements such as the confirmation of an aGvHD or cGvHD, objectively measure disease activity, allow a distinction of organ damage, should provide prognostic risk assessment, and predict responses to therapy (5). Detailed documentations on collection of specimens including excellent clinical data on patient characteristics and HSCT course, and standardized data analyses are crucial for further data processing (5). cGvHD is a multi-organ autoimmune disorder and is the major cause of non-relapse morbidity and mortality following allo-HSCT, occurring in about 50% of patients, or 13,000–15,000 patients per year worldwide that causes a plethora of comorbidities including cardiovascular, gastrointestinal (GI), liver, pulmonary, endocrine (diabetes, hyperlipidemia, thyroid and adrenal insufficiency, hypogonadism), neuropsychiatric (e.g., depression, chronic neurologic diseases), bone and joint (osteoarthritis, osteoporosis) disorders, infections (bacterial, viral, and fungal), and other more specific comorbidities (solid malignancy, obesity, and infertility) (1–5). Herein, we discuss B-cell-based, stress protein-, and microRNA (miRNA)-based biomarkers as predictors for a/cGvHD.

## Cellular Biomarkers

### The Role of T/B-Cells As Cellular Biomarkers in cGvHD

For a long time it has been known that cross-reactive allo-T cells that were immunized by environmental antigens derived from the donor are key players in the induction of GvHD. Therefore, the determination of these cells and the measurement of their cytolytic activities against host tissues have been used to determine GvHD (6). The outcome of haploidentical HSCT can be improved by the depletion of T cells from the donor graft. However, this procedure is often accompanied by graft failure and an increased incidence of GvHD, which could be overcome by megadose HSCT, injecting of pathogen-specific T cells to rebuild immunity or engineered T cells to induce suicide in case of allo-reactivity (7). The sequential infusion of regulatory T cells (Tregs) (CD4^+^/CD25^+^ and CD4^+^/CD25^−^) after HSCT, the selective *ex vivo* (photo) depletion of certain autoreactive T cell clones, the preservation of γ/δ T cells in the stem cell graft, and the selection of the best stem cells provide other options to improve GVL effects while GvHD is not increased (7). All these procedures contribute to fewer infection and toxicity rates and leukemia-related death cases.

Recent research has demonstrated that apart from T cells, B-cells also play key roles in the pathogenesis of cGvHD. Therefore, the presence of auto- and alloantibodies, elevated plasma levels of B-cell activation factor (BAFF), a cytokine of the tumor necrosis family, and an accumulation of CD19^+^CD21^low^ B-cells serve as biomarkers for GvHD. Apart from the depletion of T-cells by antibodies, the depletion of certain B-cell subpopulations might also provide a promising strategy to avoid GvHD (8–10). A delayed B-cell reconstitution with relative B-cell lymphopenia can result in downregulated B-cell counts in patients after HSCT (9–12). Low B-cell counts in the circulation may be explained in part by the insufficient production of B-cells in the bone marrow, as previously reported in patients with both, aGvHD and cGvHD (13). In contrast, a dysregulated B-cell homeostasis with persistent high BAFF levels can induce an upregulation of certain subpopulations of B-cells. In patients who do not develop cGvHD, elevated BAFF levels normalize after 6 months, whereas these remain highly elevated in patients developing cGvHD at later time points (11, 12). The observed high BAFF/B-cell ratio in patients with cGvHD suggests that during B-cell deficiency, autoreactive B-cell clones that would otherwise undergo negative selection could potentially survive due to an excess of BAFF, which in turn could possibly contribute to the pathophysiology of cGvHD (14–16). Furthermore, increased B-cell activation, aberrant B-cell signaling, and prolonged survival of activated B-cells have been found to be associated with cGvHD (17).

Perturbation of B-cell homeostasis can be associated with elevated or decreased numbers of different B-cell subpopulations during cGvHD (8, 11, 12, 16, 18, 19). Greinix and colleagues reported on elevated relative numbers of CD19^+^CD21^low^ B-cells in patients with active cGvHD compared to those without cGvHD in a retrospective study on 70 patients (8). In addition, CD19^+^CD21^low^ B-cell counts higher than 15% in patients with active cGvHD were found to be significantly associated with the presence of severe opportunistic infections (8). Furthermore, the memory B-cell compartment showed significantly lower relative and absolute numbers of both, non-class-switched CD19^+^CD27^+^IgD^+^ and class-switched CD19^+^CD27^+^IgD^−^ memory B-cells. This observed perturbation of circulating B-cell subpopulations could be useful for assessing cGvHD activity and for identifying cGvHD patients at risk for severe infectious complications (8).

Kuzmina and colleagues investigated whether the number of CD19^+^CD21^low^ B-cells could predict the outcome of extracorporeal photopheresis (ECP), which is used as one option for an immunomodulatory treatment of cGvHD (19). ECP non-responders had significantly higher (*p* = 0.02) relative numbers of CD19^+^CD21^low^ B-cells (mean = 22%) in the peripheral blood prior to the start of ECP compared to patients achieving a complete response (CR) (mean = 8%) and partial response (mean = 16%) after 6 months of ECP therapy. These data suggest that CD19^+^CD21^low^ B-cell counts could serve as a predictive cellular biomarker. Moreover, CR patients had significantly lower relative numbers of CD19^+^CD21^low^ B-cells 6, 12, and 21 months after start of ECP compared to non-responders, confirming that CD19^+^CD21^low^ B-cells could be potential cellular biomarkers for objective response assessment in cGvHD (19).

CD19^+^CD21^low^ B-cells were further investigated in cGvHD patient cohorts in the context of impaired humoral immunity defined by increased or decreased serum immunoglobulin G (IgG) levels (11). cGvHD patients with hypogammaglobulinemia had significantly decreased absolute numbers of CD19^+^ B-cells with elevated percentages of CD19^+^CD21^low^ B-cells and transitional CD19^+^CD21^int-high^CD38^high^IgM^high^ B-cells compared to cGvHD cohorts with normogammaglobulinemia or hypergammaglobulinemia, respectively. Furthermore, cGvHD patients with hypogammaglobulinemia also had a significant reduction of non-class-switched CD19^+^CD27^+^IgD^+^ and class-switched CD19^+^CD27^+^IgD^−^ memory B-cells compared to the other two cohorts. Of note, cGvHD patients with hypergammaglobulinemia presented with significantly higher BAFF/B-cell ratios and frequently had significantly more autoantibodies present compared to the hypogammaglobulinemia cohort (11). Taken together, these data suggested that B-cell subpopulations could indicate different pathogenic mechanisms involved in cGvHD and might allow a distinction between immunodeficiency and autoimmunity (11, 16).

Investigation of B-cell subpopulations in the context of specific organ involvement by cGvHD revealed significantly decreased absolute and relative numbers of CD19^+^ B-cells in patients with newly diagnosed lung involvement seen as bronchiolitis obliterans syndrome (BOS) (4, 16). The prognosis of BOS is poor, and therefore, the identification of patients at an early disease stage when they may have an improved response to immunosuppressive therapy is a major clinical challenge. Kuzmina and colleagues observed that patients with newly diagnosed BOS had significantly increased relative numbers of CD19^+^CD21^low^ B-cells (25.5 versus 6.6%, *p* < 0.0001) and BAFF/CD19^+^ ratio (0.18 versus 0.02 ng/10^3^ CD19^+^ B-cells, *p* = 0.007) compared with patients without cGvHD (16). Asymptomatic patients with a drop in pulmonary function tests and NIH-defined changes in high-resolution CT scan could be distinguished from patients without cGvHD due to significantly higher CD19^+^CD21^low^ B-cells and ratio of BAFF/CD19^+^ B-cells. Interestingly, relative numbers of CD19^+^CD21^low^ B-cells were significantly elevated both at onset of BOS as well as in patients with long-lasting BOS not responding to immunosuppressive treatment confirming a role of CD19^+^CD21^low^ B-cells as cellular biomarkers for objective diagnosis of lung involvement as well as continued cGvHD activity during the course of therapy.

In a large prospective study with 227 patients, Greinix and colleagues performed serial analyses starting on day 100 after HSCT to investigate CD19^+^CD21^low^ B-cells as diagnostic and predictive cellular biomarkers in patients with newly diagnosed cGvHD (18). Higher relative numbers of CD19^+^CD21^low^ B-cells, analyzed on day 100 after HSCT and compared to time-matched controls without cGvHD, were significantly associated with later development of cGvHD independently of clinical parameters (23.5 versus 15.2%, *p* = 0.004). Furthermore, significantly higher percentages of CD19^+^CD21^low^ B-cells were also associated with first diagnosis of cGvHD (18.3 versus 9.9%, *p* = 0.001) (18). Although their exact biological functions still need to be elucidated, it is known that CD19^+^CD21^low^ B-cells express inflammatory tissue-homing receptors, such as CXCR3 reflecting an increased capacity to home to inflammatory sites (20). Suryani and colleagues reported the production of significantly higher amounts of anti-nuclear autoantibodies by CD19^+^CD21^low^ B-cells compared to other B-cell subpopulations such as naïve CD19^+^CD10^−^CD21^high^CD27^−^ and memory CD19^+^CD27^+^IgD^−^ B-cells, respectively (21). However, recent findings suggest that CD19^+^CD21^low^ B-cells share the phenotype of anergic B-cells and do not proliferate in response to normal B-cell stimulation factors (22, 23).

Recently, regulatory B-cells (Bregs) have been shown to be involved in the pathogenesis of cGvHD (22, 24). Khoder and colleagues reported an enrichment of interleukin-10 (IL-10)-producing Bregs within memory CD19^+^IgM^+^CD27^+^ and transitional CD19^+^CD24^hi^CD38^hi^ B-cells in healthy individuals (25). In patients with cGvHD, Breg cells were found to be decreased and thus less likely to produce IL-10 compared to healthy donors and patients without cGvHD (25). Another study reported a decrease of CD24^hi^CD27^+^ B-cells and IL-10-producing CD24^hi^CD27^+^ B-cells in patients with active cGvHD (26). Moreover, de Masson and colleagues observed increased CD24^−^CD38^hi^ plasmablast frequencies, but decreased IL-10-producing plasmablasts in patients with active cGvHD compared to ones without cGvHD (22). Since CD24^hi^CD27^+^ B-cells and plasmablasts are among the most elevated cellular subsets within the Breg cell compartment, these observations could further support a possible role of Breg cells in the pathogenesis of cGvHD (22). Whether these novel cellular subpopulations could serve as biomarkers of active cGvHD, however, has to await further studies on well-defined patient cohorts including time-matched controls without cGvHD.

CD4^+^CD25^+^Foxp3^+^ Tregs have an indispensable role in the maintenance of tolerance after allo-HSCT. Poor reconstitution of Tregs after HSCT correlated with subsequent development of cGvHD (27–29). Furthermore, patients with long-lasting cGvHD are known to have a deficiency of Tregs in the circulation (26, 27). These findings led to therapeutic interventions aiming at enhancing Treg cell numbers by administration of low-dose interleukin-2 and thus, suppressing clinical manifestations of cGvHD (26). Based on these results, Tregs can be considered to be diagnostic and predictive cellular biomarkers of cGvHD.

Regarding other T-cell subpopulations, an increase in CCR7^−^CD45RA^+^CD8 T-cells that are effector memory T-cells and a decrease of CCR7^+^CD45RA^+^ naïve T-cells as well as CCR7^+^CD45RA^−^ central memory T-cells has been reported in patients with cGvHD (30). The authors speculated whether their findings were the consequence of prolonged alloantigen exposure or interleukin-15 (IL-15) stimulation since patients with cGvHD reportedly have elevated IL-15 levels and effector memory T-cells can be generated by IL-15 stimulation. Yamashita and colleagues also observed a significantly higher percentage of CCR7^−^CD62L^low^CD4^+^ effector memory T-cells in patients with cGvHD compared to ones without cGvHD or healthy donors (31). Furthermore, a preponderance of effector memory CD4^+^ T-cells relative to CCR7^+^CD45RA^−^ central memory T-cells was observed in severe cGvHD by these investigators. In view of the small patient numbers in these single-center studies, confirmation and validation of these findings is required prior to using these T-cell subpopulations as diagnostic cellular biomarkers of cGvHD.

Greinix and colleagues recently reported that CD4^+^CD45RA^+^ naïve T-cells and CD4^+^CD45RA^+^CD31^+^ T-cells were significantly increased in patients with newly diagnosed cGvHD compared to a time-matched patient cohort without cGvHD (18). Furthermore, this T-cell subset when measured prospectively on day +100 after HSCT was also significantly elevated in patients subsequently developing cGvHD compared to patients never experiencing cGvHD. Besides these T-cell subpopulations, CD3^+^CD56^+^ NKT cells were significantly increased on day +100 after HSCT in patients subsequently developing cGvHD and thus, could also serve as a predictive cellular biomarker.

## Soluble Biomarkers

### 70-kDa Heat Shock Protein Family (HSP70) As a Biomarker for Tumors and GvHD

Members of the HSP70 family are known to play an important role in transport, folding, and unfolding of proteins and also in the induction of immune responses (32–38). The major stress-inducible Hsp70 has been found to be upregulated in many different diseases including inflammation, autoimmunity, and tumors (32, 39). Hsp70 is also found on the cell surface of many tumor types including leukemic cells via a tumor-specific lipid anchorage (40). Furthermore, it was shown that membrane Hsp70 positive, viable tumor cells are able to secrete Hsp70 into the circulation in lipid vesicles, termed exosomes (33, 41). Therefore, exosomal Hsp70 serves as a tumor-specific biomarker for viable tumor mass (42). Functionally, extracellular HSPs, either alone or in combination with antigenic peptides, play important roles in the induction of inflammatory immune responses (34, 35, 43, 44). Apart from lipid-bound Hsp70, Hsp70 can be released by necrotic and inflamed tissues as a free molecule. This means that the major stress-inducible Hsp70 is secreted into the circulation either by living tumor cells in lipid vesicles, presumably tumor exosomes (41), or as a free molecule by dying necrotic and/or inflamed tissues. In cGvHD, inflammatory responses can occur in skin (77%), lung (50%), mouth (63%), liver (58%), eye (54%), joints (32%), GI (20%), and genital tract (16%). Therefore, free Hsp70, antibodies directed against Hsp70, mRNA levels might provide potential novel molecular biomarkers to diagnose and predict the onset of inflammation in cGvHD. Apart from members of the HSP70 family other stress proteins are discussed as potential markers for GvHD.

In a rat skin explant model, Novota et al could demonstrate that the two major stress-inducible genes Hsp70-1 and Hsp70-2 were upregulated. Therefore, elevated mRNA levels were associated with the grade of graft-versus-host reactions (GvHR) (45). Moreover, in the study by Kim et al., it was shown that a polymorphism in Hsp70-hom plays an important role in the prognosis of patients who received a sibling HLA-matched allo-HSCT (46). Thus, Hsp70-hom gene polymorphism might also serve as a prognostic marker for GvHD (47). A subsequent study by Bogunia-Kubik et al showed that patients who were homozygous for the A allele of the Hsp70-hom +2,663 SNP presented more frequently with grade II and IV toxic lesions and aGvHD compared to patients with different genotypes (48).

Other studies clearly demonstrated an involvement of the Hsp70 protein levels in the pathogenesis of GvHD. In a rat GvHD model that was induced by the injection of DA parental lymphoid cells into irradiated (LEW × DA)F1 rats, it was shown that the expression levels of Hsp70 were significantly increased in spleen and lymph nodes 7 and 14 days after induction of the disease (49). At later time points of the disease, the Hsp70 levels dropped to levels that were comparable to that of untreated control animals.

Different results exist with respect to HSP70-specific antibodies in the circulation in a rat model and human patients. In a rat model, elevated levels of antibodies (IgM, IgG2a, and IgG2b) directed against HSP70 have been found to be associated with the onset of symptoms that were associated with the development of GvHD (50), whereas in pediatric patients, this association was not detected after allo-HSCT (51). In subsequent human studies however, elevated anti-HSP70 and anti-HSP90 antibody levels were found to be associated with the development of a/cGvHD (52).

An association of the severity of GvHD disease and the expression of Hsp70 might be explained by the immunomodulatory activity of Hsp70. To date, several studies reported an involvement of Hsp70 in the production of pro-inflammatory cytokines such as TNF-α, IL-1, IL-6, and IL-12 and the secretion of nitric oxide (NO) and C-C chemokines by dendritic cells, monocytes, and macrophages (53, 54). Moreover, Hsp70 can activate intracellular signaling cascades that influence immunoregulatory functions of immune cells through binding of either free or lipid-bound Hsp70 to specific cell surface receptors such as Toll-like receptors 2 and 4 (TLR2/4), scavenger receptor CD36, low-density lipoprotein receptor-related protein CD91, C-type lectin receptor LOX-1, scavenger receptor SR-A, and CD40 (35). Through the induction of pro-inflammatory cytokines, Hsp70 contributes to the pathogenesis of autoimmune and various chronic inflammatory diseases (55–58). Thus, Hsp70 can serve as a damage-associated molecular pattern that activates the host’s adaptive and innate immune system by initiating alloimmunity (59). Another mechanism of the immunoregulatory activity of Hsp70 is based on its peptide-binding capacity. Exogenous Hsp70 can chaperone and cross-present antigens and cargo them to the antigen-presenting cells and thus elicit adaptive immune responses (44, 60). Therefore, it is not too surprising that Hsp70 is frequently upregulated in allografts, which in turn results in the progression of disease and allograft rejection (61–63).

Presumably, the modulation of Hsp70 levels might provide a therapeutic option for improving the outcome of GvHD. Previously, Oh et al showed, in a skin graft model, in which Hsp70 (Hsp70.1 gene) knock-out (KO) mice were used either as a donor or recipient, the importance of Hsp70 in acute allograft rejection (64). Allogeneic cells derived from Hsp70 KO mice were shown to induce lower rejection rates in recipients than those of Hsp70 wild-type animals. Therefore, the application of reagents that are able to silence Hsp70 expression may provide a promising strategy to reduce the risk for GvHD. The reduction of HSP70 levels by the administration of 15-deoxyspergualin (DSG), an immunosuppressive agent that binds to a constitutively expressed member of the 70-kDa heat shock protein family, has been shown to significantly reduce GvHD-associated mortality (65). DSG treatment reduced HSP70 levels in spleen and lymph nodes, inhibited the anti-HSP70 antibody production, and reduced the serum levels of IL-2, IFN-γ, TNF-α, and IL-10 (64).

In conclusion, monitoring levels of HSP70 protein and/or anti-HSP70 antibody levels in the serum after HSCT might serve as a diagnostic tool to predict the onset of GvHD. In addition, genetic or pharmacological modulation of the HSP70 expression may have a therapeutic potential in the treatment of GvHD.

### Protein Markers in Body Fluids for Diagnosis of aGvHD

Serum, plasma (66–68), and saliva have been analyzed for prediction or diagnosis of aGvHD and cGvHD (69–71). A surface-enhanced laser desorption/ionization (SELDI) mass spectrometer was used to analyze plasma/serum in patients with and without GvHD post-HSCT by the group of Barrett in 2006 at the National Heart, Lung, and Blood Institute, National Institute of Health (NIH). They used SELDI to identify proteins/peptides differentially excreted into plasma (69–71). Another group from the NIH analyzed salvia samples using SELDI, no further data have been reported for either study. Therefore, plasma proteomics may be a more promising approach. The group of Ferrara and colleagues have intensively studied the serum/plasma proteomic approach using enzyme-linked immunosorbent assays to detect the presence of several proteins in the serum/plasma of patients post allo-HSCT (66–68). Among other markers, they have described plasma markers for GI aGvHD (68), regenerating islet-derived protein 3a (Reg-3a) and suppression of tumorigenicity (ST2) as diagnostic markers for aGvHD and survival after HSCT. Reg-3a was tested in samples from 1,014 HSCT patients from three transplantation centers and Reg3a concentrations were threefold higher in patients at onset of GI GvHD than in all other patients and correlated with lower GI GvHD. Reg3a concentrations at time of GvHD onset predicted response to therapy at 4 weeks, 1-year NRM, and 1-year survival (*p* ≤ 0.001). In a multivariate analysis, advanced clinical stage, severe histologic damage, and high Reg3α concentrations at GvHD diagnosis independently predicted 1-year NRM. The combination of Reg3a with clinical stage and histologic grade of GvHD can be used to improve risk stratification of patients.

The described biomarkers have been tested in two different centers (67, 68). To fulfill the criteria of a reliable biomarker, the biomarkers have to be defined in a test set, confirmed in a first validation set and then validated in a multicenter validation trial (5), as biomarkers need to be robust under different clinical conditions and prophylaxis strategies.

The group of Levine and Ferrara has recently achieved this goal for the so-called Ann Arbor Score of aGvHD, which relies on three biomarkers (Reg3 alpha, ST2, and sTNFR1). If this score is high at the time of onset of GvHD, it strongly predicts day 28 treatment response and 6-month NRM irrespective of center-specific strategies (72).

An international Mount Sinai Acute GvHD International Consortium (MAGIC consortium) has been recently founded, which prospectively monitors clinical data and samples from more than 1,000 pts receiving allogeneic SCT/year and now allows to develop biomarker score-based treatment stratification at the time of onset of GvHD (73).

None of the serum/plasma markers described to date can predict aGvHD development, but they can help to diagnose aGvHD, especially GI aGvHD and to define prognosis at a very early time point where clinical presentation fails to allow exact prediction. Earlier biomarker scores (e.g., at day 7) after HSCT are currently developed and hopefully will allow stratification of preemptive treatment in the future. Taken together, the proteomic monitoring of patients holds promise for early diagnosis as well as risk stratification of patients.

Serum/plasma biomarker development for cGvHD is yet not at the state of prospective multicenter monitoring. Chemokine (C-X-C motif) ligand 9 (CXCL9), suppression of tumorigenicity 2 (ST2), osteopontin, and soluble BAFF levels are candidates among others (74–77). In particular, recent work by Yu et al has described a biomarker panel for cGvHD using quantitative proteomic profiling by high-resolution tandem mass spectrometry. Pooled plasma from patients with and without cGvHD at matched time points posttransplant were tested in a discovery set and two independent validation cohorts.

The matrix metalloproteinase 3 (MMP3) in the plasma correlates with BOS, a serious complication after allo-HSCT. An area under the receiver-operating characteristic (ROC) curve for MMP3 indicated a value of 0.77 (78).

Another biomarker that may be linked to cGvHD or to the maintenance of remission is the presence of H-Y specific antibodies that relate to a minor antigen mismatch. These antibodies appear approximately 4–12 months after HSCT predominantly in male patients who were transplanted with female donor grafts. In the presence of alloantibodies, the cumulative incidence of cGvHD reached 89% at 5 years after HSCT compared with only 31% in the absence of H-Y antibodies. However, the cumulative incidence of relapse reached 48% at 5 years in the absence of H-Y antibodies compared with 0% in the presence of H-Y alloantibodies. Therefore, the authors concluded that antibody responses to H-Y antigens were associated with maintenance of disease remission in gender-mismatched HSCT. In a follow-up research project, the same group demonstrated that H-Y antigen-binding B-cells developed in male recipients of female hematopoietic cells that were associated with cGvHD (79). Of note, B-cells specific for the dominant H-Y epitope, DEAD box protein (DBY-2) appeared in significantly higher frequency in the circulation 6 months after HSCT in individuals who developed cGvHD later and thus, may predict cGvHD (79, 80). However, this single center study requires validation in larger prospective clinical studies to allow firm conclusions.

### Biofluid miRNAs As Biomarkers for Graft-versus-Host Disease

Within the last decade, circulating short single-stranded miRNA has been identified in human plasma, serum, and also in urine (81, 82). Interestingly, these miRNAs were found to be resistant to RNase, boiling, changes in pH, extended storage, and freeze-thaw cycles (83). Functionally, miRNA molecules are considered as one of the major groups of translational regulators with the ability to regulate differentiation of blood cells and immune functions (84, 85). The stability of miRNA in the circulation can be attributed to three major RNase protection mechanisms: miRNAs can be bound to protective proteins, such as nucleophosmin 1 (NPM1) and/or Agonaute 2 (Ago2) (86–88), miRNA can form complexes with lipid or lipoproteins including high-density lipoprotein and low-density lipoprotein, and miRNA can be encapsulated in extracellular lipid vesicles, such as exosomes (89, 90). The complex of miRNA and these proteins and/or vesicles allows the selective export of miRNAs into the circulation and the protection of them within the extracellular environment (86). Circulating miRNAs have been shown to act as robust biomarkers for a various diseases, including autoimmune conditions, such as rheumatoid arthritis and systemic lupus erythematosus and tumors (91). A number of studies have also assessed the association between circulating miRNAs and the development of GvHD, with promising results (92–96).

Circulating miRNAs are robust and can be detected in biofluids by minimal invasive methods using relatively simple and accurate technologies. Furthermore, circulating miRNAs may offer advantages over protein-based biomarkers as they are lower in complexity, conserved among clinically relevant species, expressed specifically in different tissues or biological stages and easily measured using common laboratory techniques (97).

**Figure 1:**
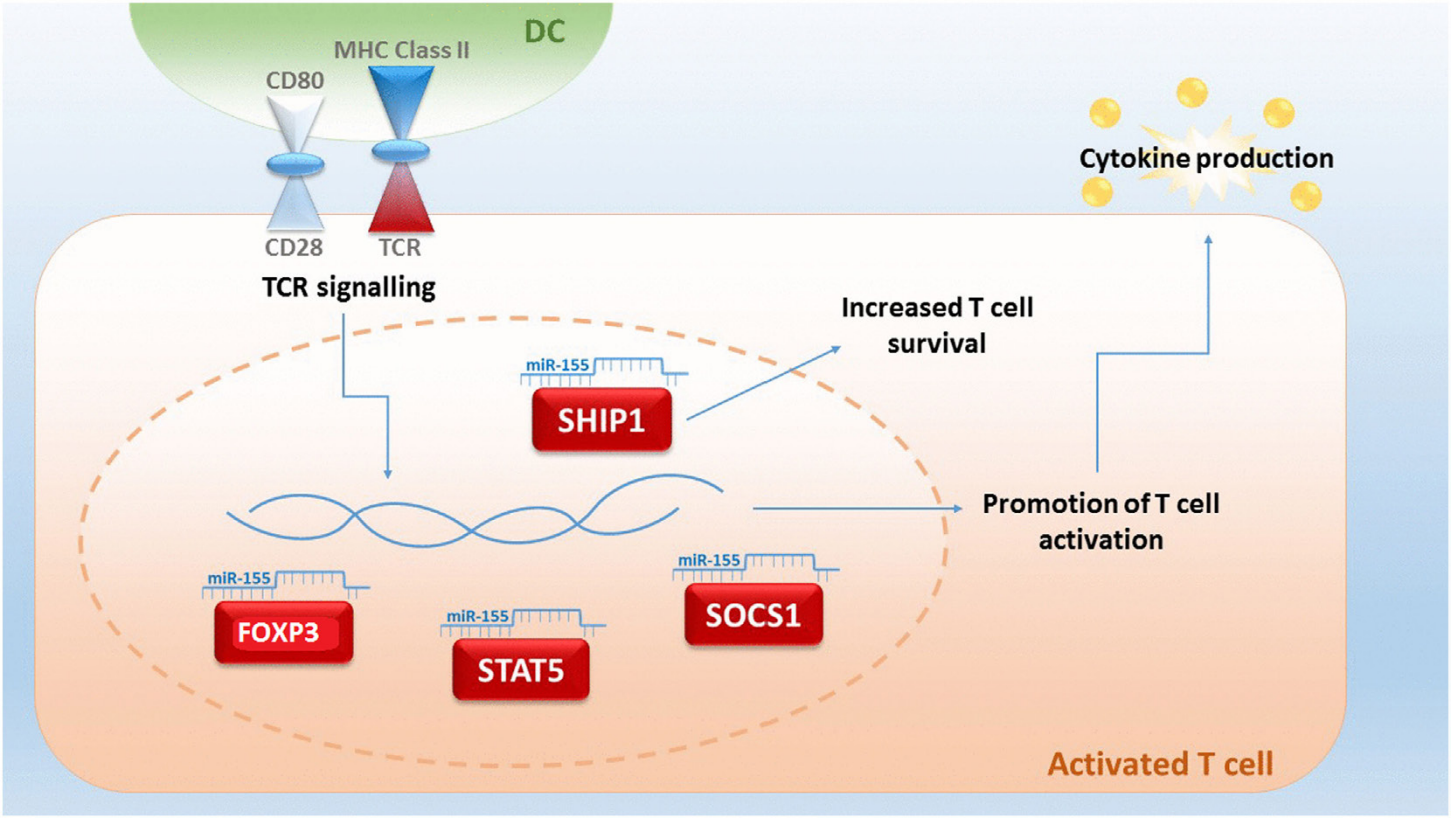
**An upregulated expression of microRNA (miRNA)-155 in T-cells is indicative for lethal acute graft-versus-host disease (aGvHD) in mice**. miR-155 plays a central role in fine-tuning the immune response, and expression can be triggered by T cell receptor (TCR) signaling. miRNA-155 regulates FOXP3, which is important for the survival of T regulatory cells and influences the targets signal transducer and activator of transcription factor 5 (STAT5) and suppressor of cytokine signaling 1 (SOCS1). Targeting of 3,4,5-triphosphate 5-phosphatase 1 (SHIP1) by miRNA-155 results in an increased T-cell survival by an upregulated production of IFN-γ (93, 104–108).

Although miRNA studies in relation to GvHD are still in their infancy, miRNA-155 was the first miRNA to be associated with aGvHD (98). miRNA-155 expression is upregulated in T cells of mice with severe aGvHD after allo-HSCT and a reduction of miRNA-155 results in a decreased severity of aGvHD and a prolonged survival of mice (99). miRNA-155 is encoded within the B cell integration cluster and is important for the regulation of acute inflammation and innate immunity (98). Expression of miRNA-155 can be activated by inflammatory mediators, such as IFN-α/γ and TNF-α (100), and Ceppi et al proposed that it functions as part of the negative feedback loop controlling the secretion of inflammatory cytokines by LPS-induced DC activation (101). Thus, miRNA-155 is pivotal in fine-tuning of the immune response (Figure 1). Physiologically, miRNA-155 is upregulated during T cell differentiation, plays a role in CD4^+^ and CD8^+^ T cell-mediated immunity and promotes the development of T cells, including Th17 and Treg subsets. Mice with a germ-line depletion of miRNA-155 have a normal lymphocyte development but defective T and B cell immunity (102, 103). Within Tregs, miRNA-155 targets forkhead box P3 (FoxP3), which regulates *in vivo* Treg survival (104). miRNA-155 also directly targets the IL-2 signaling protein suppressor of cytokine signaling 1 (SOCS1), whereby miRNA-155 deficiency in Tregs results in increased SOCS1 expression (96). This, in turn, leads to impaired activation of signal transducer and activator of transcription factor 5 (STAT5) phosphorylation and IL-2 receptor signaling (105). miRNA-155 is also a key regulator of CD8^+^ T cell responses via SOCS1, where a deficiency of miRNA-155 results in defective STAT5-mediated cytokine signaling (106). In CD4^+^ T cells, miRNA-155 targets phosphatidylinositol 3,4,5-triphosphate 5-phosphatase 1 (SHIP1) for downregulation, which normally functions to suppress Th1 responses and T cells by modulating IFN-γ production (102, 107).

In a seminal study in 2012, Ranganathan et al showed that miRNA-155 is upregulated in T-cells from mice developing GvHD following HSCT (98). Moreover, the use of miRNA-155 inhibitors decreased disease severity and prolonging survival (98). In addition, the group found increased miRNA-155 expression in small and large bowel biopsies compared to normal bowel tissue in patients with GI aGvHD (98). However, these studies focused on miRNA expression within tissue biopsies, and more recently, several groups have sought to exploit the biomarker potential of circulating miRNAs in the context of GvHD. Although the presence of miRNAs has been established in a number of biological fluids (103), studies to date have concentrated on serum and plasma fractions of the blood.

A comprehensive report published by Xiao et al (92) in 2013 used a high-throughput qRT-PCR-based array to profile the expression of 345 miRNAs in the plasma of patients with aGvHD compared to those with no GvHD. They employed a discovery and training cohort to identify a final signature of four miRNAs (miRNA-423, miRNA-199-3p, miRNA-93*, and miRNA-377). These four miRNAs were able to predict aGvHD 6 weeks post-HSCT, prior to the onset of symptoms. The model was significant in ROC analysis (AUC = 0.76, *p* < 0.001) and was also associated with disease severity and poor overall survival (92). The miRNAs were shown to be specific for aGvHD, as they were not detected in the plasma of lung transplant or non-transplant sepsis patients. This study highlighted the potential of biofluid miRNAs as independent markers for prediction, prognosis, and diagnosis of GvHD.

Although miRNA-155 has been previously associated with the development of GvHD, it was not included in the final model proposed by Xiao et al. While expression was significantly upregulated in the plasma of aGvHD patients, levels were the lowest among the miRNA candidates identified (92). However, a recent study by Xie et al focusing on serum showed that miRNA-155 was significantly upregulated in aGvHD patients (*p* = 0.003) and also correlated with disease severity (*p* < 0.001) (93). Furthermore, expression was elevated in cGvHD patients compared to those who did not develop the disease (*p* = 0.005); however, levels were not sufficient to distinguish between aGvHD and cGvHD (*p* = 0.96) (93). Although this study showed promise for the inclusion of miRNA-155 as a GvHD biofluid biomarker, the study was restricted to one cohort of Han Chinese population (*n* = 64). Thus, the results need to be validated in independent prospective cohorts.

A small study by Sang et al confirmed elevated miRNA-155 plasma levels in patients with aGvHD, and additionally reported upregulation of miRNA-92b, while miRNA-150 and miRNA-181 were significantly downregulated (95). Interestingly, levels of the miRNAs were also altered prior to disease onset, highlighting their biomarker potential for predicting incidence (95). However, the difference in miRNA-181 was most pronounced, with no variation observed in control patients compared to reduced levels of expression in 19/22 (86%) patients prior to disease development (95). The group also demonstrated miRNA-181 to act as an effective predictor of aGvHD in a murine allo-HSCT model (95); however, larger cohorts are required in order to validate these findings.

More recently, Wang et al performed a study focusing on miRNA-586 expression in the plasma of HSCT patients (96). miRNA-586 was significantly upregulated in patients who developed aGvHD compared to no aGvHD as early as 7 days post-HSCT; however, expression was influenced by infection that reduced the significance of the association (96). As infections are common in early post-HSCT and can make distinguishing a diagnosis of aGvHD more challenging, it is important to validate the findings in larger studies as well as to elucidate the biological role of miRNA-586 in infections. Thus, although these results shown promise, miRNA-586 may play a greater role as a clinical biomarker to differentiate aGvHD from infectious complications.

In a separate study, Gimondi et al focused on lymphoma patients receiving MUD allo-transplants to profile the plasma using qRT-PCR for global miRNA expression (94). Assessing samples collected 28 days post-HSCT, 113 miRNAs were detected in all samples and of these, 27 could collectively discriminate between aGvHD versus no aGvHD and miRNA-194 and miRNA-518f were significantly upregulated in the patients who later developed the disease (94). The authors did not detect differential expression of miRNAs identified by Xiao et al (92), and there was no validation cohort included in the investigation. Thus, although results showed potential for the identification of aGvHD biomarkers, additional confirmatory studies are required.

Although circulating miRNAs as biomarkers for GvHD show great promise, these studies are still in their infancy and few overlapping targets between reports have been identified. A summary of presently known miRNAs for a/cGvHD have been presented in Table 1. Much work is needed to validate the findings in independent cohorts that reflect the heterogeneity in conditioning and prophylaxis regimens employed by different clinical centers. Moreover, collaboration between groups will allow for the standardization of protocols and technologies, which may greatly influence the reproducibility of findings and is a likely explanation for the lack of concordance in results to date. For blood studies, the choice of serum or plasma needs to be considered, with slightly more biomarker studies currently focusing on serum over plasma, while few whole blood or PBMC studies have been performed (108). Indeed, it has been proposed that profiles from isolated PBMC may yield information on the immune status of the disease, while the serum and plasma levels reflect the disease-dependent secretion and expression of miRNAs (108). The effect of hemolysis and platelet contamination as well as the choice of anti-coagulant should also be considered (109–111). Selection of the miRNA detection platform and normalization as well as the RNA isolation methodology employed has also been shown to affect results. Indeed, analytical variables have a huge potential to bias results, and this is particularly dependent on the normalization methods used (112). Some level of correction may be achieved by spiking in synthetic miRNAs (113); however, this approach can correct for technical variation such as the efficiency of RNA isolation and reverse transcription, but it will not account for intrinsic biological variation (114). Thus, the choice of normalization controls and/or global normalization becomes integral to the data analysis strategy. With regard to the miRNA detection platform, although qRT-PCR is a commonly used approach, variations on the fluorescent molecules including TaqMan and Sybr-Green, as well as the development of newer techniques including NanoString and Next-generation sequencing, have increased heterogeneity. This is important, as variation in results have been observed depending on the analysis platform, partly attributed to the difficulty in distinguishing small molecules at low abundance with high sequence homology (115).

**Table 1:** **Role of different microRNAs (miRNAs) in acute and chronic graft-versus-host disease (a/cGvHD)**.

MicroRNA	Findings	Reference
miR-155	↑ in bowel tissue in aGvHD	(98)
miR-155	↑ in plasma in aGvHD and cGvHD	(93)
miR-155	↑ in plasma in aGvHD	(95)
miR-423	↑ in plasma in aGvHD	(92)
miR-199-3p	↑ in plasma in aGvHD	(92)
miR-93*	↑ in plasma in aGvHD	(92)
miR-377	↑ in plasma in aGvHD	(92)
miR-92b	↑ in plasma in aGvHD	(95)
miR-150	↓ in plasma in aGvHD	(95)
miR-181	↓ in plasma in aGvHD	(95)
miR-586	↑ in plasma in aGvHD	(96)
miR-194	↑ in plasma in aGvHD	(94)
miR-518f	↑ in plasma in aGvHD	(94)

Despite these considerations, it is expected that over the next few years, as the number of circulating miRNA biomarker studies increases, specific miRNA patterns for GvHD will be proposed and validated in the clinic. Although studies to date have focused on fractions of the blood, the potential for discovery of miRNA signatures in other fluids, including urine, is attractive. Indeed, initial data promise that miRNA signatures may be identified to predict the incidence of GvHD prior to onset, the severity of disease, to distinguish GvHD from other complications, and even to differentiate between aGvHD and cGvHD, particularly in relation to late onset aGvHD. Given the heterogeneity of transplantation protocols employed throughout different clinics, miRNA models appropriate to different transplant protocols may also be possible. Further investigation of validated miRNA signatures will allow the impact of miRNA dysregulation on the pathogenesis of GvHD to be studied, and conclusively, prospective investigations assessing the outcome of treatments selected by miRNA status will confirm their prognostic strength. Ultimately, the aim will be to diagnose GvHD and outcome before clinical symptoms manifest, allowing earlier introduction of therapy, tailored treatments, and reduced mortality and morbidity outcomes.

## Urine Proteomics Based on Capillary Electrophoresis and Mass Spectrometry (CE-MS)

### Proteomics for Prediction of aGvHD after HSCT

Proteomics of tissues or body fluids has gained significant importance over the last 10 years.

For example, urinary proteome-based classifiers, developed for aGvHD prediction, were first described in 2004 (116) and adapted in 2007 (117) and additional prospective evaluation was provided in 2014 (118). The urinary proteome profiling for aGvHD is done on prospectively collected urine samples from patients undergoing HSCT. The urine is collected prior to HSCT and conditioning, weekly after HSCT until day +35 and afterward biweekly until day +100. Urine can be frozen at −20°C until preparation of the sample for analyses using CE-MS (119–122). An extension to include patients with cGvHD was carried out in 2008; thus, samples were collected bimonthly after day +100 or at diagnosis of cGvHD (119, 123).

**Figure 2:**
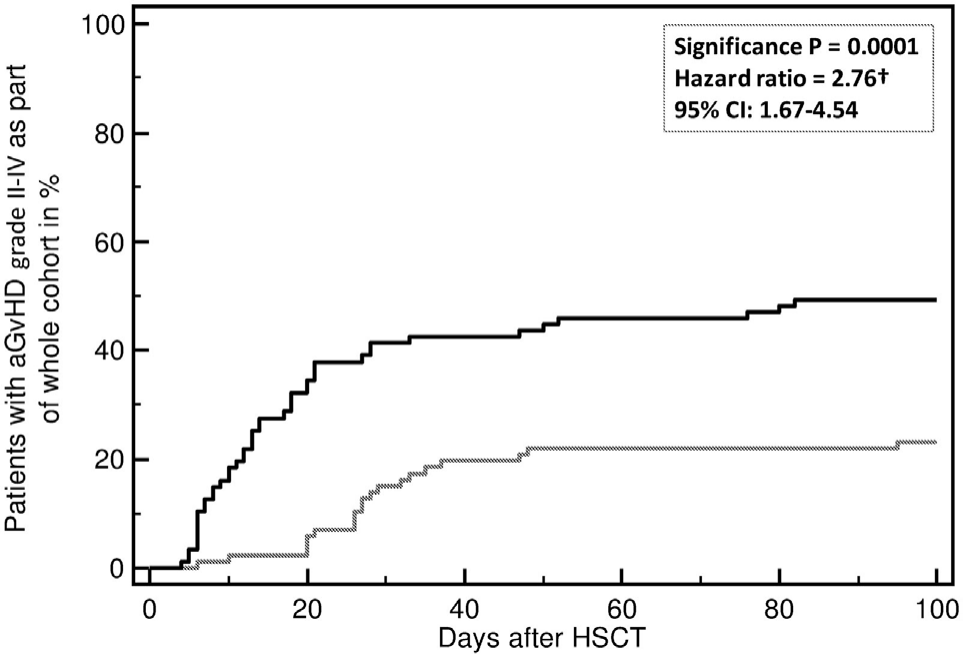
**Incidence of developing an acute graft-versus-host disease (aGvHD) grades II–IV**. Incidence of aGvHD grades II–IV is predicted by aGvHD_MS17 positivity (*p* = 0.0001) when compared to patients without aGvHD_MS17 pattern positivity. Shown is the percentage (%) of patients with aGvHD II–IV with (black line) or without aGvHD_MS17 positivity over time [days after allogeneic hematopoietic stem cell transplantation (allo-HSCT)] (gray line).

Acute and chronic GvHD are currently diagnosed on clinical parameters defined in NIH Consensus (2, 124, 125) and German Consensus Conferences (126–128). Prediction of aGvHD using the investigator-independent, unbiased proteomic classifier aGvHD_MS17 was done on more than 500 patients in at least 4 centers in Germany and UK and resulted in stable prediction of aGvHD about 7–10 days prior to clinical diagnosis or biopsy positivity (118, 119, 123, 129). The classifier aGvHD_MS17 consists of 17 peptides, 10 of which have been sequenced to date (117–119). The identified peptides are various fragments of collagen (indicating disturbances in metabolism of collagen and/or early organ damage) and fragments of fibronectin, beta-2-microglobulin (β2M), and CD99, an activation marker of T-cells. Multivariate regression analysis showed that aGvHD_MS17 positivity was the highest predictive parameter for aGvHD development (*p* < 0.0001, Figure 2).

## Future Perspectives

Numerous candidates for biomarkers are currently available, which include plasma and serum markers, cell-based markers such as B-cell subpopulations, and T follicular helper cells as regulators of B-cell immunity in determining cGvHD in patients (130), miRNAs, different stress proteins, or proteomic approaches using plasma, serum, or urine. Proteomic mass spectrometry analysis coupled with computational biology approaches will further lead to the identification of novel biomarkers for GvHD such as BOS in the lung (131). Recently, the diversity of the intestinal microbiome as analyzed by 16 s rRNA sequencing of stool microbiota (131–134) and the assessment of bacterial metabolites (135) very early after transplantation has identified patients at high risk of lethal complications. On the other hand, biomarkers can also be used to tailor immunosuppressive therapies in transplanted patients and thus might predict the severity of GvHD.

The main challenge remains to integrate all these candidates and to validate them as biomarkers that are valid in multiple centers independent of the center’s strategy of prophylaxis, and consortia on prospective testing of these markers together with clinical data collection as shown for the MAGIC consortium are urgently needed. The expectations have to be defined clearly, do we want to predict outcome in general or treatment response for aGvHD or cGvHD or do we want to make a diagnosis as precise as possible (e.g., for GI GvHD, for cGvHD) and the current standards and endpoints for each of these parameters have to be defined separately (e.g., d28 response for aGvHD, 2- or 3-month response for cGvHD, 6 months NRM for aGvHD, composite endpoints of GvHD-free and relapse-free survivals) (136).

New candidates have to be integrated in this process step by step, and bio-mathematical approaches are needed to define those markers that give true additive information. In addition, integration of older markers has not been performed (e.g., calprotectin in stool samples for GI GvHD (137), the poor man’s biomarker albumin for GI GvHD) (138, 139), and more importantly, testing biomarkers against clinical predictors or markers of outcome is needed. For aGvHD, factors indicating poor prognosis are the extent of organ involvement and number of involved organs. For cGvHD, the type of onset and simultaneous occurrence of thrombocytopenia should be addressed. It might well be that some of the markers behave as highly sensitive and specific markers due to the correlation with extent and type of involved organ and the specific pathophysiology, which might be reflected by the correlation with clinical parameters (140).

## Author Contributions

MJ, MS, PM, and JO equally contributed to the manuscript. MJ, MS, PM, JO, and RC wrote the manuscript and prepared the figures. AD, HG, EH, EW, and GM outlined and proof-read the manuscript.

## Conflict of Interest Statement

The authors declare that the research was conducted in the absence of any commercial or financial relationships that could be construed as a potential conflict of interest.
